# Aspirin in COVID-19: Pros and Cons

**DOI:** 10.3389/fphar.2022.849628

**Published:** 2022-03-10

**Authors:** Rana Zareef, Marwa Diab, Tala Al Saleh, Adham Makarem, Nour K. Younis, Fadi Bitar, Mariam Arabi

**Affiliations:** ^1^ Pediatric and Adolescent Medicine Department, American University of Beirut Medical Center, Beirut, Lebanon; ^2^ Faculty of Medicine, American University of Beirut Medical Center, Beirut, Lebanon; ^3^ Brigham and Women’s Hospital-Harvard Medical School, Boston, MA, United States; ^4^ Pediatric Department, Division of Pediatric Cardiology, American University of Beirut Medical Center, Beirut, Lebanon

**Keywords:** COVID-19, SARS-CoV-2, coronavirus, aspirin, salicylic acid

## Abstract

Since its emergence, the COVID-19 pandemic has been ravaging the medical and economic sectors even with the significant vaccination advances. In severe presentations, the disease of SARS-CoV-2 can manifest with life-threatening thromboembolic and multi-organ repercussions provoking notable morbidity and mortality. The pathogenesis of such burdensome forms has been under extensive investigation and is attributed to a state of immune dysfunction and hyperinflammation. In light of these extraordinary circumstances, research efforts have focused on investigating and repurposing previously available agents that target the inflammatory and hematological cascades. Aspirin, due to its well-known properties and multiple molecular targets, and ought to its extensive clinical use, has been perceived as a potential therapeutic agent for COVID-19. Aspirin acts at multiple cellular targets to achieve its anti-inflammatory and anti-platelet effects. Although initial promising clinical data describing aspirin role in COVID-19 has appeared, evidence supporting its use remains fragile and premature. This review explores the notion of repurposing aspirin in COVID-19 infection. It delves into aspirin as a molecule, along with its pharmacology and clinical applications. It also reviews the current high-quality clinical evidence highlighting the role of aspirin in SARS-CoV-2 infection.

## Introduction

The latest pandemic caused by the novel SARS-CoV-2 virus has led to the emergence of coronavirus disease 2019 termed COVID-19. The disease first appeared in Wuhan, China in December 2019 as an outbreak of atypical pneumonia (Younis et al., 2020; Rana O; Zareef et al., 2020; Tsang et al., 2021; Younis et al., 2021). The virus has ever since spread at an unpreceded pace, exhausting the global health sector and endangering the economies (Arthi and Parman, 2021; Padhan and Prabheesh, 2021). SARS-CoV-2 belongs to the Coronaviridae family. Although the majority of Coronaviridae family members are implicated in mild upper respiratory tract illness, SARS-CoV-2 has caused a wide range of more serious illnesses (Morens et al., 2020). Overall, most COVID-19 patients exhibit mild to moderate disease (Landete et al., 2020). However, a small percentage of patients may display severe sickness and are placed at greater risk of experiencing mortality and morbidity (Landete et al., 2020). Studies have shown that some factors including obesity, older age, cardiovascular comorbidities, pre-existing pulmonary condition, and chronic kidney disease, among other factors, are associated with increased risk of hospitalization, mechanical ventilation and mortality (Attaway et al., 2020; Feng et al., 2020; Klang et al., 2020; Williamson et al., 2020; Wu and McGoogan, 2020; Phelps et al., 2021). In these cases, the disease has beleaguered multiple organ-systems imparting significant irreversible damage. Unfortunately, the cardiovascular system is also embattled, with subsequent substantial complications has been reported (Magadum and Kishore, 2020; Zareef et al., 2020). Besides, a high rate of coagulopathy has been described in patients infected with COVID-19, suggesting a critical COVID-19 induced thromboembolic event. Such events are major cardiovascular complications and are associated with increased mortality (Zareef et al., 2020; Zhou et al., 2020; Aktaa et al., 2021). Studies have highlighted an astonishing rate of venous thromboembolism and pulmonary embolism in COVID-19 patients reaching 42 and 17% respectively in severe cases (Wu et al., 2021). Arterial thrombotic events at various sites including coronaries, brain, and extremities have also been described (Mehra et al., 2020; De Roquetaillade et al., 2021).

Vaccines developed against SARS-CoV-2 virus have shown effective reduction in transmission rate as well as the pattern of hospitalization, ventilation and mortality, as evident by the clinical trials (Polack et al., 2020; Voysey et al., 2021). However, even with the large-scale global vaccination programs, the virus has attained several remarkable mutations and produced new variants such as B.1.1.7, P.1, B.1.351, B.1.427, P.3, B.1.429, B.1.526, and B.1.617.2 (Baden et al., 2021; Chookajorn et al., 2021; Vasireddy et al., 2021; Voysey et al., 2021). This is particularly problematic as emerging variants might acquire the ability to transmit rapidly, and cause more severe disease, while escaping the host immune system (Vasireddy et al., 2021). They might as well challenge the previously proven vaccine efficacy (Bernal et al., 2021). Due to the rapid rise of events during the pandemic, and the high mortality and morbidity rates, the quest for therapeutic strategies has been ongoing. In this manner, drug repurposing has constituted an attractive mean for fighting the current crisis. Several regimens have been tried including steroids, azithromycin, ivermectin, hydroxychloroquine, tocilizumab, baricitinib, antivirals among others (Santos et al., 2020; Younis et al., 2020; Younis et al., 2021b). In light of the evidence of thromboembolic events and the noticeable hyperinflammatory state, several studies and investigators have suggested a possible role for aspirin in treating COVID-19 disease (Mohamed-Hussein et al., 2020). This paper discusses the potential therapeutic role of aspirin in COVID-19 disease through dissecting its pharmacology, cellular targets, clinical uses, as well as the current high quality clinical evidence.

## Pharmacology of Aspirin

Salicylic acid (Aspirin) is produced and administered via different routes in various doses and forms (Arif and Aggarwal, 2021). The usual therapeutic range of serum salicylate concentration is 10–30 mg/dl (0.7–2.2 mmol/L). Indeed, the dosing of aspirin is crucial as it dictates its mechanism of action. Traditionally, anti-thrombotic effects are achieved at low doses (75–81 mg/day), analgesic and antipyretic effects are achieved at intermediate doses (650 mg–4 g/day), while aspirin at high doses (between 4 and 8 g/day) is effective as an anti-inflammatory agent (Pillinger et al., 1998).

Aspirin intoxication can occur after ingesting 10–30 g in adults and as little as 3 g in children. Most patients exhibit signs and symptoms of intoxication if the serum concentration level of salicylate exceeds 40–50 mg/dl (2.9–3.6 mmol/L) (Hill 1973).

### Pharmacokinetically

Acetylsalicylic acid is in general rapidly and completely absorbed by the gastrointestinal tract following oral administration (Awtry and Loscalzo, 2000). However, absorption may also be variable depending on several factors including the route of administration, the dosage, the rate of tablet dissolution, gastric pH, gastric contents, and emptying time (INC, 2017). It is mainly absorbed in the stomach and small intestine. The plasma concentration of salicylate peaks between 1 and 2 h following administration. It gets distributed to all body tissues shortly after administration, mainly to peritoneal, spinal, synovial fluids, milk, saliva, liver, kidneys, heart, and lungs. It is also known to cross the placenta (DrugBankonline, 2005). Aspirin is hydrolyzed in plasma to salicylic acid and its levels become undetectable 4–8 h after administration. The liver is the main site of metabolism for salicylate, although other tissues may also be involved. It then gets eliminated by the kidneys via glomerular filtration and tubular excretion processes (INC, 2017). An entire dose needs around 48 h for the salicylate to be completely eliminated. The half-lives of ASA versus salicylate is 15 min versus 4 h respectively, while the clearance rate of ASA is variable depending on several factors (DrugBankonline, 2005).

### Pharmacodynamically

The pharmacodynamic aspect of aspirin is unique, as it doesn’t interact with any surface or intracellular receptors. It exerts its activity through non-specific irreversible acetylation of molecules. The acetylation process instigates alterations at the level of macromolecules, and accordingly adjusts the function of the proteins. Due to the irreversibility of such modification, the duration of activity depends on the turnover rate of the target molecule irrespective of aspirin plasma concentration (Schrör, 2016).

## Mechanism of Action of Aspirin

Aspirin is one of the most commonly used drugs worldwide (Vane and Botting, 2003; Zhou et al., 2014). It is an anti-inflammatory, anti-pyretic, analgesic, and anti-platelet drug. Aspirin exerts its major activity by inhibiting the cyclooxygenase enzyme (COX), which exhibits two forms: COX-1 and COX-2 (Patrono et al., 2001) (Patrono et al., 2001). Subsequently, it blocks the conversion of arachidonic acid into prostaglandins and thromboxane, collectively called prostanoids. Its activity expands to target several other structures circumventing a set of inflammatory and thrombotic events.

### Anti-Inflammatory Activity

**FIGURE 1:**
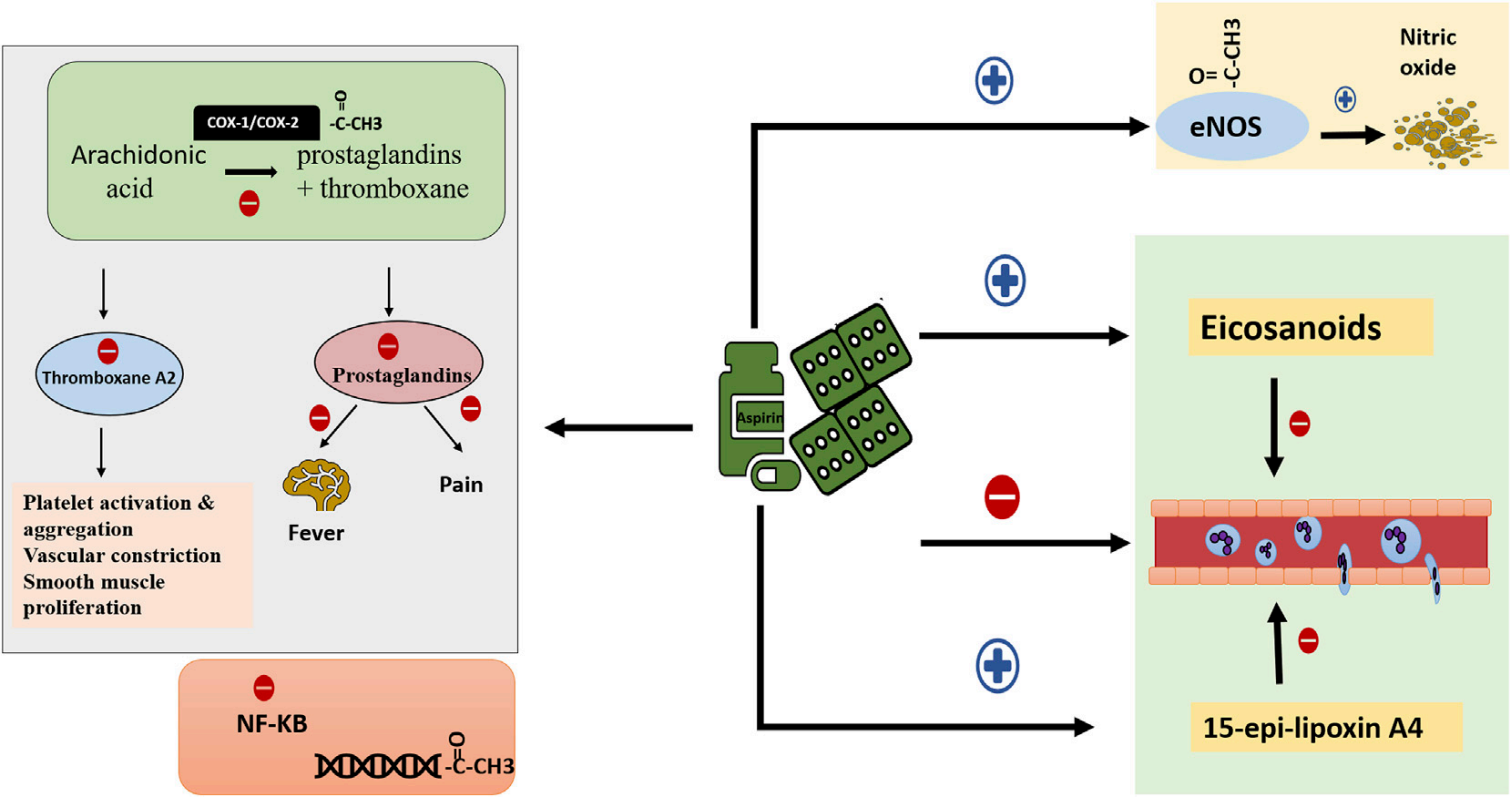
Mechanism of action of aspirin. Aspirin possesses several targets through which it exerts its activity. First, it inhibits prostanoids synthesis thus employing anti-thrombotic, anti-inflammatory, anti-pyretic and analgesic effect. In addition, it acetylates multiple cellular proteins hence affecting DNA transcription and expression. It also constrains NF-KB production, limiting its pro-inflammatory effect. Furthermore, aspirin enhances the synthesis of eicosanoids and 15-epi-lipoxin A4. Combining all together, aspirin impedes PMNs interaction with platelets and endothelium, PMNs chemotaxis, adhesion, and migration. Finally, it acetylates and activates eNOS to maintain vascular homeostasis. (NF-KB: nuclear factor kappa B; PMNs: polymorphonuclear cells; eNOS endothelial nitric oxide synthase).

Aspirin exerts its anti-inflammatory property through several mechanisms (Figure 1). At intermediate and high concertation, aspirin non-selectively acetylates and inhibits the activity of COX-1 and COX-2, hampering the biosynthesis of prostanoids and their subsequent inflammatory outcome. Indeed, COX-2 expression is induced by inflammatory cytokines, hormones, and growth factors, and it plays a role in cancer, acute stress, inflammation, and infection (Chiang and Serhan, 2009). Aspirin also interferes with innate immunity through the inhibition of thromboxane A2 production. Thromboxane A2 facilitates platelet-polymorphonuclear (PMNs) cells interaction and the subsequent migration of PMNs to the areas of inflammation. This also takes place at low doses of aspirin (Patrono et al., 1980; Cooper et al., 2004). Similarly, at low concentrations, aspirin stimulates the synthesis of certain eicosanoids that terminates the trafficking of PMNs (Claria and Serhan, 1995). Low dose aspirin also inhibits leukocyte adhesion and migration by stimulating the synthesis of 15-epi-lipoxin A4. 15-epi-lipoxin A4, known as aspirin-triggered 15-epi-lipoxin A4 (ATL), pathway alters leukocyte/endothelium interactions and limits leukocyte extravascular accumulation (Perretti et al., 2002; Serhan, 2002). In fact, the anti-inflammatory effect of aspirin doesn’t stop at altering the biosynthesis of prostaglandins and thromboxane, it also interferes with various cellular pathways to intrude on the inflammatory response. Several studies have shown that aspirin disturbs intracellular signaling pathways including nuclear factor-kappa B (NF-K B) (Kopp and Ghosh, 1994; Grilli et al., 1996; Yin et al., 1998). NF-KB plays a role in the inflammatory response. Aspirin reduces the production of NF-KB, and at the same time inhibits the breakdown of its inhibitor (Kopp and Ghosh, 1994). Besides, aspirin exerts a protective effect at the cellular level as an anti-oxidant by induction of heme oxygenase-1 during inflammatory states (Grosser et al., 2003). At higher concentrations and over longer periods, aspirin can nonspecifically acetylate other proteins (Vane and Botting, 2003). Remarkably, one study highlighted the role of aspirin in gene regulation through acetylation of histones (Sabari et al., 2017). Endothelial nitric oxide synthase (eNO) is another target for aspirin. When aspirin acetylates the enzyme, it stimulates nitric oxide release thus maintaining vascular homeostasis (Taubert et al., 2004). Aspirin likewise acts as an anti-pyretic and analgesic agent. Prostaglandins potentiate the effect and sensitivity of pain receptors and other substances like histamine and bradykinin (Patrono et al., 2001). A decrease in prostaglandins production reduces pain and inflammation. Similarly, aspirin inhibits the production of brain prostaglandin E1 which is a potent fever-inducing agent, thus acting as an antipyretic (Vane, 1976).

### Anti-Platelet Activity

Aspirin is a well-known potent anti-thrombotic (Figure 1). At low doses, the acetyl group of aspirin binds to serine 530 residue of COX-1 and irreversibly inhibits its activity (Funk et al., 1991). Therefore, prostaglandin H2 synthesis is inhibited (Patrono, 1994). Prostaglandin H2 is a substrate used by the enzyme thromboxane-A-synthase to produce thromboxane A2. Thromboxane A2 is a strong pro-thrombotic molecule that is synthesized and released by platelets; it stimulates platelets activation and aggregation (FitzGerald, 1991). It also promotes vascular constriction and smooth muscle proliferation. This inhibitory effect is irreversible. The synthesis of new thromboxane A2 depends on the synthesis of new platelets, a process occurring at a rate of 10% daily (Di Minno et al., 1983).

## Clinical Uses of Aspirin

The medicinal history of aspirin dates back to more than 3,500 years ago, where the old Egyptians and Sumerians used the willow bark as anti-pyretic and analgesic agent. As medicine progressed, the first precursor of aspirin, salicylate, was described in 1763 by Reverned Stone as antipyretic, while aspirin was first described in 1897 by Felix Hoffman (Montinari et al., 2019). Today, aspirin is a well-known and widely used drug. It represents a famous well-established antipyretic and analgesic agent. It is extensively used in cardiovascular diseases, mainly in the acute settings of myocardial infarction, unstable angina, ischemic stroke, and for secondary prevention of recurrent coronary artery disease (Framework, 1998; De Gaetano, 2001; Members; Gibbons et al., 2003; Younis et al., 2021c) (Awtry and Loscalzo, 2000; Members; Antman et al., 2004; Pan et al., 2019). Perhaps, the most common off-label use of aspirin is for the primary and secondary prevention of atherosclerotic disease. However, the protective value of aspirin has not been well established in healthy individuals with no risk or previous occlusive events. (Collaboration, 2002; Dai and Ge, 2012). Aspirin is also the drug of choice for prophylaxis in revascularization surgeries including coronary bypass surgery (Members, Eagle et al., 2004; Smith et al., 2006). While being initially widely used, aspirin’s use in rheumatic diseases such as rheumatoid arthritis has declined for two reasons: 1) its anti-inflammatory properties are established at relatively high doses, and 2) the desired effects are reached at lower doses with more efficacy with non-salicylate nonsteroidal anti-inflammatory drugs (NSAIDs) (Csuka and McCarty, 1989). Despite that, aspirin is still part of the treatment Kawasaki disease (Rife and Gedalia, 2020). Aspirin was also proved to be beneficial in preventing preeclampsia, and more ongoing trials are exploring the role of aspirin in reducing the risk of colorectal cancer (Roberge et al., 2018) (Dubé et al., 2007). In pediatrics, aspirin is used in patients with congenital heart disease and recently in treating multisystem inflammatory syndrome in children (Abani et al., 2021; Abi Nassif et al., 2021).

The use of aspirin is not without adverse effects. Increased risk of bleeding is possible, mainly secondary to decreased levels of thromboxane A2 (Arif and Aggarwal, 2021). In addition, aspirin, just like all NSAIDs, can potentially cause gastritis and ulcers, as cyclooxygenase is essential in maintaining the gastrointestinal mucosa (Awtry and Loscalzo, 2000). A rare side effect is tinnitus at high doses. Aspirin metabolite, salicylic acid, can alter cochlear nerve function, but tinnitus usually resolves after drug discontinuation (Edward et al., 2021). Reye’s syndrome, which encompasses liver failure and encephalopathy, is associated with aspirin use in children [(MD) 2017]. Given this, aspirin is not generally used in kids except in the case of Kawasaki disease.

## Aspirin and COVID-19

COVID-19 complications have been linked to an immune dysregulation syndrome accompanied by cytokine storms (Coperchini et al., 2020; Mehta et al., 2020). This severe inflammation is known to trigger the coagulation cascade and inhibit fibrinolysis, disrupting blood homeostasis. On the other hand, COVID-19 has been found to infect vascular endothelial cells leading to endotheliitis (Varga et al., 2020). Endothelial inflammation and von-Willebrand exposure to sub-endothelial collagen, in turn, precipitate thrombus formation manifesting as arterial, venous, and microvascular thromboembolic events. Aspirin, with its anti-inflammatory and antithrombotic properties, could therefore protect against severe forms of COVID infection. High dose aspirin is in fact used in MIS-C for its anti-inflammatory properties. Aspirin is also commonly used in atherosclerotic CVD to stabilize diseased arterial endothelium. Its anti-platelet properties are mediated through COX-1 inhibition preventing platelet aggregation, while its anticoagulant properties stem from factor 8 activation inhibition. Acetylsalicylic acid also stimulates fibrinolysis and modifies thrombus architecture (Mekaj et al., 2015). Aspirin, through its pleiotropic effects, was therefore naturally hypothesized to prevent COVID’s multifactorial complications and is the subject of the following review.

### Pros

**FIGURE 2:**
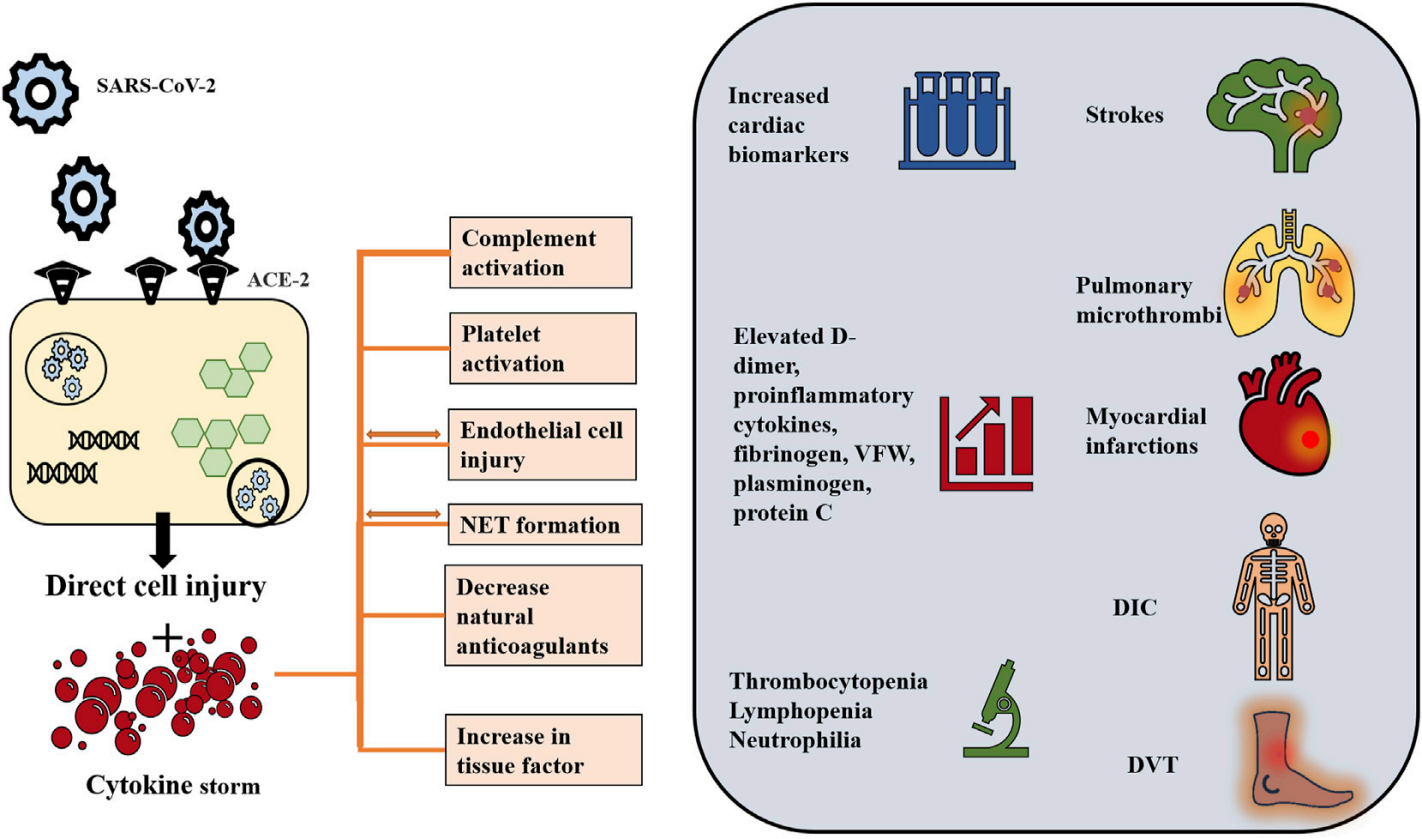
COVID-19 induced hyperinflammatory and hypercoagulable states. Although the pathogenesis of COVID-19 induced coagulopathy has not been fully elucidated, interplay among immune dysregulation, hyperinflammation and thrombosis is proposed. SARS-CoV-2 virus, via its spike protein, interacts with the ACE-2 receptor to enter the cell through endocytosis. Endothelial cells as well as respiratory cells have high expression level of ACE-2. Inside the cell, the virus releases its genetic material and replicates using the cellular machinery. The viral effect is suggested to take place through two mechanisms: 1) Direct viral injury and 2) indirect cytokine-mediated injury. The viral cytopathic effect is implicated in direct damage and apoptosis of the host cell thus contributing to endothelitis. In turn, endothelial damage triggers platelet activation and aggregation. At the same time, the virus drives intense inflammation and immune dysregulation. It suppresses the lymphocytic activity and activates macrophages and polymorphonuclear cells, thus generating pro-inflammatory cytokines including IL-1, IL-2, IL-6, IL-10, IL-17, IL-18 and TNF-α, and leading to cytokine storm in severe illness. TF production, release of VWF and the initiation of coagulation cascade are triggered by cytokines and the injured epithelium. The cytokine storm also induces activation of complement system which contributes to coagulopathy by activating platelets, and increasing production of fibrin and thrombin. The cytokine storm is also associated with NETs, which in turn promotes VWF and TF activity and disables tissue factor inhibitor and thrombomodulin, therefore induing inflammation and microvascular thrombosis. These cellular processes are reflected on laboratory values, which ae usually remarkable for a combination of prolonged prothrombin time, normal to mildly prolonged activated partial thromboplastin time, thrombocytopenia, elevated D-dimer level, fibrinogen, fibrinogen degradation products, VFW, plasminogen, protein C, and factor VIII. Clinically, the hyperinflammatory response and endothelial dysfunction affect both venous and arterial systems. Venous thromboembolism includes pulmonary embolism and deep venous thrombosis. Arterial thromboembolism including myocardial injury and strokes have been also reported. Consumptive coagulopathy and ultimately DIC is also observed in critically ill patients (Lippi et al., 2020a; Iba et al., 2020b; Lippi et al., 2020b; Goshua et al., 2020; Guan et al., 2020; Levi and Thachil 2020; Mehta et al., 2020; Middleton et al., 2020; Tang et al., 2020; Varga et al., 2020; Zhang et al., 2020; Zhou et al., 2020; Tan et al., 2021). ACE-2: angiotensin converting enzyme-2; IL: Interleukin; VWF: von Willebrand factor; NET: neutrophil extracellular traps; TF: Tissue factor; DIC: disseminated intravascular coagulation.

Pathologic examination of lung tissue in patients who died due to COVID-19 infection and its complications revealed morphological aspects of acute respiratory distress syndrome including diffuse alveolar damage, intra-alveolar edema, inflammatory infiltration by mononuclear and multinucleated cells, and vascular damage (Xu et al., 2020; Yao et al., 2020; Batah and Fabro, 2021). Further pathological examination revealed the formation of immune and fibrin microthrombi leading to intracapillary thrombosis (Colling and Kanthi, 2020; Batah and Fabro, 2021). Remarkably, it is suggested that such changes, specifically the edema and inflammatory infiltrates, develop before the pneumonia symptoms (Tian et al., 2020). Add to these the hyperinflammatory and hypercoagulable state (Figure 2), marked by laboratory aberrations namely increasing D-dimer and fibrinogen and decreasing platelet count with more severe disease (Lin et al., 2021). From these repercussions of the infection stems the utility of antiplatelet and anticoagulant agents specifically aspirin.

Because aspirin is a chronic medication for many patients, studies at first investigated the effect of chronic aspirin use on the course of COVID-19 infection (Table 1). Osborne’s retrospective study included 35,370 patients with and without active aspirin prescription before acquiring SARS-CoV2 (Osborne et al., 2021). Aspirin users had a significantly decreased risk of mortality by 32% at 14 and 30 days after infection. After adjusting to confounding covariates [age, gender, comorbidities, and the Care Assessment Needs (CAN) 1-year mortality score] and propensity score matching, mortality dropped from 6.3 to 2.5% with aspirin use at 14 days and from 10.5 to 4.3% at 30 days in the propensity matched cohorts.

**TABLE 1:** High quality clinical studies showing evidence of beneficial aspirin role in COVID-19 disease.

Author(s)	Country	Date	Study design	Dose	Mortality	Mechanical ventilation	Other outcomes
Osbourne et al.	United States	Feb-21	Retrospective database review	NA	Pre-existing aspirin use was associated with lower mortality at 14 and 30 days (adjusted OR: 0.38) Propensity matched cohort: drop in 14-day mortality from 6.3 to 2.5% and a drop in 30-day mortality from 10.5 to 4.3%		
Kow and Hasan	United States, United Kingdom, China, Italy, Germany	Apr-21	Meta-analysis	NA	Significantly reduced risk of a fatal course of COVID-19 with the use of aspirin in patients with COVID-19 relative to non-use of aspirin (pooled OR = 0.50 (0.32–0.77); pooled HR = 0.50 (0.36–0.69)		
Chow et al.	United States	Apr-21	Multicenter retrospective observational cohort	Low dose	In-hospital initiation of aspirin was independently associated with reduced in-hospital mortality (adjusted HR, 0.53, *p*-value = 0.02)	Aspirin was independently associated with a reduced risk for mechanical ventilation (adjusted HR, 0.56 (0.37–0.85), *p-value* = 0.007)	Aspirin was associated with a reduction in the risk of ICU admission (adjusted HR, 0.57 (0.38–0.85), *p-value* = 0.005)
Liu et al.	China	Feb-21	Single-center retrospective cohort	Low dose	In-hospital aspirin initiation had significantly lower 30-day and 60-day mortality compared to the non-aspirin group		No significant difference in the viral duration time (time from 1st positive PCR to 1st negative PCR) between the two groups
Meizlish et al.	United States	Apr-21	Multicenter retrospective	Low dose	In-hospital, aspirin initiation was associated with a lower cumulative incidence of in-hospital death, on multivariate regression and propensity score matching (HR = 0.52)		
Aghajani et al.	Iran	Apr-21	Retrospective cohort	NA	Aspirin (HR = 0.753 [0.573–0.991], *p*-value = 0.043) was associated with decreased risk of in-hospital mortality	16.07% of aspirin users and 90 13.74% of nonusers needed mechanical ventilation (*p*-value = 0.324)	Length of hospital stay was significantly longer in patients who received aspirin (*p*-value < 0.001)

Other studies investigated in-hospital aspirin use irrespective of pre-infection use. A retrospective observational cohort study by Chow et al., studied the severity of the disease in patients who received aspirin within the first day of admission or a week before admission (Chow et al., 2021). Patients in both groups had similar vital signs and lab tests, except fibrinogen level, which was significantly lower in patients receiving aspirin. On initial crude analysis, there was no difference in in-hospital mortality between the two groups in spite of the improvement in other outcomes and no difference in rates of bleeding and overt thrombosis. However, after adjusting to age, sex, race, body mass index (BMI), comorbidities and home beta blocker use, patients receiving aspirin had reduced risk of mechanical ventilation, intensive care unit (ICU) admission and in-hospital mortality, these results persisted on subgroup analysis. In addition, sensitivity analysis was performed by stratifying patients relative to the timing of aspirin use: started in the first 24 h, in the 7 days prior to admission, or both; this analysis showed lower rates of ICU admission in patients who started aspirin in the first 24 h compared to the others.

Low in-hospital mortality was also deduced in other studies assessing different populations (Haji Aghajani et al., 2021; Liu et al., 2021). In those studies, patients were receiving aspirin during their hospitalization not withstanding prior chronic aspirin use, had similar baseline vital signs, inflammatory and infectious markers, and medications used during hospitalization.

To be able to assess the efficacy of aspirin in an acute COVID-19 setting, Meizlish et al. dissected the use of anticoagulants and antiplatelets in COVID-19 patients with documented abnormalities in D-dimer and fibrinogen levels (Meizlish et al., 2021). At first, propensity matched cohorts comparing patients receiving aspirin vs. patients receiving no aspirin were considered, adjusting to multiple factors including physicians’ tendency to administer aspirin to critically ill patients. Multivariate analysis showed a lower cumulative incidence of in-hospital death in the aspirin cohort (Meizlish et al., 2021). After May 18, they released a recommendation to administer aspirin for all patients hospitalized for COVID-19, which showed similar favorable outcomes.

While considering the favorable outcomes that these studies revealed, one should consider many factors. All of these included studies were retrospective in nature. Some studies had an adequate number of patients but from a homogeneous sample enlisted in the Veterans Health Administration, which makes the results generalizable only to this population (Osbornes et al., 2021). Additionally, most studies failed to distinguish between the efficacy of chronic and acute aspirin uses. None of them have specified whether chronic aspirin use is superior to using aspirin only in the acute setting, and vice versa. Yet, the abovementioned studies indeed took into consideration the baseline factors that might affect patients’ clinical course as well as eventual morbidity and mortality, including underlying conditions, demographics, vital signs, and lab values at presentation and others. Specifically, in Haji Aghajani et al., the initial bivariate analysis revealed higher in-hospital mortality in aspirin users (Haji Aghajani et al., 2021). After adjusting to the aforementioned factors, aspirin was found to be protective of mortality, although aspirin users were admitted for longer and needed more time on the mechanical ventilator.

A meta-analysis done by Kow and Hassan included 12 studies, six of which investigated antiplatelet use in COVID-19, and six others investigating aspirin use (Kow and Hasan, 2021). The pooled analysis showed a significant benefit to aspirin use in protecting the patients from a fatal course of COVID-19. To note, this benefit was not seen when considering antiplatelet use, suggesting that maybe the other effects of aspirin, specifically its anti-inflammatory properties, account for the favorable results.

### Cons

Alongside publications supporting the use of aspirin in COVID-19 patients, there is also antipodal literature arguing against it (Table 2).

**TABLE 2:** High quality clinical evidence displaying negative role for aspirin in COVID-19 disease.

Author(s)	Country	Date	Study design	Mortality	Mechanical ventilation	Other outcomes
Yuan et al.	China	Jan-21	Retrospective database review	No difference in mortality between CAD patients taking and not taking aspirin	No difference in need of mechanical ventilation between the 2 groups	No difference in severe disease, inflammatory markers, liver and kidney function and lung imaging between patients taking and not taking aspirin pre-hospitalization
Sahai et al.	United States	Dec-20	Retrospective database review	Neither aspirin nor NSAIDs affected mortality. They were associated with increased risk of MI, CVA, or VTE		
Salah and Mehta	United States, China, Iran	Mar-21	Meta-analysis	Mortality was not associated with the use of aspirin in patients with COVID-19 (RR 1.12, [0.84, 1.50])		
Son et al.	South Korea	Jul-21	Case control	Mortality was not associated with the use of aspirin. Adjusted OR = 0.92 (0.46–1.84)		No correlation between prior aspirin use and COVID-19 complications. Adjusted OR = 1.06 (0.66–1.69)
Abdelwahab et al.	Egypt	Jul-21	Retrospective cohort		No correlation between prior aspirin use and mechanical ventilation Adjusted OR = 1.095, *p*-value = 0.932	Decreased risk of thromboembolic events with prior aspirin use. Adjusted OR = 0.163, *p* = 0.02
Pan et al.	United States	May-21	Retrospective cohort	Mortality was not associated with the prior use of anti-platelets. Adjusted OR = 1.13 (0.70–1.82)		No correlation between prior anti-platelet use and the composite outcome (high oxygen need, invasive ventilation and death). Adjusted OR = 0.98 (0.65–1.46)
Tremblay et al.	United States	Jul-20	Retrospective cohort	Mortality was not associated with the prior use of anti-platelets. HR = 1.029 (0.723–1.466)	No correlation between prior anti-platelet use and mechanical ventilation. HR = 1.239 (0.807–1.901)	No correlation between prior anti-platelet use and either survival time, time to mechanical ventilation or hospital admission
Russo et al.	Italy	May-20	Retrospective cohort	In-hospital mortality was not associated with the prior use of anti-platelets. Adjusted RR = 0.51 (0.21–1.15) *p*-value = 0.110		No correlation between prior anti-platelet use and ARDS upon admission. Adjusted RR = 0.58 (0.38–1.14), *p*-value = 0.165
Banik et al.	Germany	Nov-20	Retrospective cohort	No correlation between prior anti-platelet use and the composite endpoint death or transfer for ECMO. Adjusted OR = 2.25 (0.0456–270)	No correlation between prior anti-platelet use and the need for mechanical ventilation. Adjusted OR = 0.781 (0.0253–17.0)	Prior anti-platelet use correlated with a positive chest CT. Adjusted OR = 12.1 (1.41–167), *p*-value = 0.0354 prior use of anti-platelet did not correlate with the length of hospital stay
Horby et al.	United Kingdom, Indonesia, Nepal	Jun-21	RCT	28-day mortality was not associated with aspirin treatment. RR = 0.96 (0.89–1.04) *p* = 0.35	Mechanical ventilation need was not associated with aspirin treatment. RR = 0.96 (0.9–1.03)	Rate of discharges before 28 days was slightly higher among patients in aspirin arm. RR = 1.06 (1.02–1.1) *p*- value = 0.0062 median time until discharge was 8 days in aspirin users versus 9 days in non-users. There was no correlation with successful cessation of mechanical ventilation or need for renal replacement therapy
Kim et al.	South Korea	Sep-21	Retrospective cohort	Increased risk of death among patients who took aspirin within the 2-weeks prior to COVID-19 diagnosis (40%) vs. those who did not (5%) *p*-value = 0.027; however, groups were not matched for prior CAD No correlation between mortality and aspirin treatment within 2 weeks after diagnosis	Mechanical ventilation need was not associated with aspirin treatment either before (*p*-value = 0.141) or after (*p*-value = 0.173) diagnosis	People who received aspirin after diagnosis were at higher risk of needing oxygen therapy (46.7%) vs. those who did not receive aspirin (35.0%), *p*-value <0.0001. No correlation between oxygen need and aspirin use before diagnosis. No correlation between COVID infection rate and prior aspirin use. No correlation between aspirin use before or after diagnosis and ICU admission

CAD, coronary artery disease; NSAIDS, non-steroidal anti-inflammatory drugs; MI, myocardial infarction; CVA, cerebrovascular accident; VTE, venous thromboembolism; ARDS, acute respiratory distress syndrome; ECMO, extra-corporeal membrane oxygenation; ICU, intensive care unit.

The RECOVERY trial is, to date, the only published randomized clinical trial (RCT) testing the benefits of aspirin therapy in COVID-19 patients. This trial is an open-label, platform RCT that recruited 14,892 inpatients with COVID-19. In this study, 7,351 patients were randomly allocated to receive 150 mg of aspirin daily alongside usual care, while 7,541 received usual care alone. It involved 177 hospitals in the United Kingdom, two hospitals in Indonesia, and two hospitals in Nepal. Authors did not find a correlation between aspirin intake and 28-day mortality, the study’s primary outcome (rate ratio 0.96; 95% confidence interval 0.89–1.04; *p* = 0.35). There was also no significant difference in the composite outcome of mechanical ventilation or death within 28 days of admission between the two treatment arms (risk ratio 0.96; 95% CI 0.90–1.03; *p* = 0.23). On the other hand, a slightly higher but significant proportion of inpatients on aspirin was discharged alive before 28 days (75 vs. 74%; rate ratio 1.06; 95% CI 1.02–1.10; *p* = 0.0062) (Abani et al., 2021).

This study only looked at inpatients and excluded those on chronic aspirin therapy. This trial being a platform RCT, it also involved looking at many drugs simultaneously. For example, 90% of patients in this trial were taking corticosteroids while 93% were on low molecular weight heparin (LMWH). Authors suggest these high rates of antithrombotic therapy with LMWH and corticosteroid treatment must have decreased thrombo-inflammatory stimulation in the entire study population. Therefore, the treatment arm might not have significantly benefited from aspirin given that almost everyone was anticoagulated and taking corticosteroids. However, in a real-life scenario where a patient is admitted and given steroids and LMWH, it seems the addition of aspirin is not indicated and will not further improve outcomes. Aspirin might also be more beneficial among people with a higher risk of thrombosis, i.e., the patients on chronic antiplatelet therapy that were excluded from the study.

The remaining literature against aspirin use in COVID-19 disease stems from retrospective cohort and case control studies, none of which found a correlation between aspirin treatment and mortality despite adjusting for comorbidities such as cardiovascular disease and its risk factors (older age, hypertension, diabetes, hyperlipidemia, smoking … ) (Pan et al., 2019; Russo et al., 2020; Tremblay et al., 2020; Banik et al., 2021; Sahai et al., 2021; Son et al., 2021; Yuan et al., 2021). These studies looked at patients with an underlying condition and who were prescribed aspirin before testing positive for COVID-19. The bias imparted by their comorbidities should be accounted for when adjusting for risk factors, however it cannot be excluded that a confounder might have been missed in the statistical analysis. Furthermore, no correlation was found between mortality and either aspirin use in specific (Sahai et al., 2021; Son et al., 2021; Yuan et al., 2021) or antiplatelet use more generally (Pan et al., 2019; Russo et al., 2020; Tremblay et al., 2020; Banik et al., 2021). The results were also similar between inpatient populations (Pan et al., 2019; Russo et al., 2020; Banik et al., 2021; Yuan et al., 2021) and mixed ambulatory and hospitalized patients (Tremblay et al., 2020; Sahai et al., 2021; Son et al., 2021). In addition, despite combining the findings of Russo et al. and Tremblay et al. in a meta-analysis, the effect of antiplatelets on mortality was still insignificant OR = 0.65 (0.40–1.06) *p* = 0.498 for a total of 3,964 patients (Russo et al., 2020; Tremblay et al., 2020). Similarly, but in a non-COVID setting, a meta-analysis done by Liang et al. including a pooled population of 6,764 patients showed that prior aspirin use was linked with a significantly lower incidence of ARDS in at-risk patients (*p* = 0.018) but had no effect on hospital mortality (OR, 0.88; 95% CI, 0.73–1.07; *p* = 0.204; I^2^ = 0%) (Liang et al., 2020). These findings were validated by Wang et al. in their 2018 meta-analysis on the same subject (Wang et al., 2018).

Microthrombi formation plays an essential role in COVID-19 pathophysiology, aspirin use could therefore be potentially beneficial in that regard. However, findings concerning aspirin’s effects are contradictory. Sahai et al. noticed an increased risk of thromboembolic events among COVID-19 patients on chronic aspirin therapy despite adjustment for comorbidities like age, sex, smoking, hypertension, diabetes, and cardiovascular diseases (adjusted OR = 0.163, *p* = 0.005), however they failed to adjust for a history of MI, stroke or venous thromboembolism (VTE) (Sahai et al., 2021). Aspirin therapy may therefore simply be a coincidental signal of the increased baseline risk of thrombosis in these patients. However, it could also be an indication of a different mechanism of thrombosis in COVID-19. Manne et al. detected a deranged and altered platelet phenotype in SARS-COV-2 (Manne et al., 2020). While Elbadawi et al. found absolute neutrophil count to be an independent predictor of thrombotic events in patients with COVID-19 (Elbadawi et al., 2021). Several studies have also shown the role of neutrophil extracellular traps in thrombus formation (Skendros et al., 2020). These findings supporting an immunological trigger to thrombosis suggest platelets may be indirect mediators in this process and perhaps not the best direct targets for pharmacological intervention (Sahai et al., 2021).

Another retrospective trial conducted in Egypt by Abdelwahab et al. found that the risk of stroke or MI among patients on chronic aspirin therapy was significantly lower than among non-users after adjustment (*p* = 0.02), but anticoagulants were found to be even more effective (adjusted OR = 0.071, *p* < 0.001) (Abdelwahabet al., 2021). The essence of coagulopathy in COVID-19 is indeed hypothesized to be massive fibrin formation. Anti-platelets might therefore not work as well as anti-coagulants if microthrombi have more fibrin than platelets. In fact, 90% of hospitalized COVID-19 pneumonia patients have elevated D-dimer levels. D-dimer can also reach very high thresholds and it is associated with disease severity (Iba et al., 2020a).

Another studied outcome was the need for mechanical ventilation, and here too there was no clear benefit for aspirin among COVID-19 patients. Antiplatelets were not found to affect the need for or time to mechanical ventilation, nor the risk of acute respiratory distress syndrome (ARDS) upon admission among patients on aspirin or other anti-platelets (taken as primary or secondary cardiovascular prophylaxis or as treatment for thromboembolic disease) (Pan et al., 2019; Russo et al., 2020; Tremblay et al., 2020; Abdelwahab et al., 2021; Banik et al., 2021). Limitations within these studies include failure to adjust for smoking status and BMI, small sample size (Abdelwahab et al., 2021), inclusion of only inpatients (Abdelwahab et al., 2021), and lack of correction for the influence of post-admission treatments (Tremblay et al., 2020). Banik et al. were able to detect a positive effect of anti-coagulants on mechanical ventilation need but found no correlation between antiplatelets and intubation (Banik et al., 2021). Surprisingly, they found that a positive chest CT was correlated with antiplatelet intake despite adjustment (OR = 12.1 (1.41–167), *p* = 0.0354). While another retrospective cohort showed an increased risk of oxygen therapy in patients prescribed aspirin within 2 weeks after diagnosis (Kim et al., 2021); but here propensity matching failed to adjust for a history of CAD. SARS-Cov-2 was indeed found to infect pulmonary endothelial cells causing endothelial injury and thrombosis (Acosta and Singer, 2020). It also seems that an important feature of ARDS is platelet and/or neutrophil aggregation (Zarbock et al., 2006). Gongalves de Moraes et al. showed that aspirin treatment could increase neutrophil number in the bronchial alveolar fluid in a mouse model (Gongalves de Moraes et al., 1996) which could potentially explain the correlation between aspirin intake and a positive chest CT, even though this correlation had no prognostic value as noted by Banik et al.

## Conclusion

The pandemic has imparted significant burdens on the global health and economic sectors, leaving behind substantial morbidity and mortality. While the scientific community merged efforts to obtain the vaccine at an unpreceded velocity, the search for a therapeutic agent is still ongoing. Aspirin with its various molecular targets and properties has been under clinical investigations. Gathering all the high-quality clinical evidence to date, it appears that the effect of aspirin is still not clearly delineated. Despite the large number of studies exploring aspirin role in COVID-19 disease, the evidence is still premature. Almost all studies are retrospective in nature, and many fail to consider baseline clinical status that might eventually alter the outcomes measured. More studies are needed to better define recommendations of clinical practice. The anti-inflammatory and anti-platelet properties of aspirin are appealing, yet future studies have to pledge to more objective designs. Multi-center placebo-controlled high-quality randomized clinical trials with plainly outlined baseline characteristics and outcomes are urgently needed to evaluate the efficacy of aspirin.
